# A multicenter, real‐world study on effectiveness and safety of first‐line modified PD‐1 inhibitors with chemotherapy in advanced non‐small cell lung cancer (aNSCLC) with drive gene‐negative

**DOI:** 10.1002/cam4.7024

**Published:** 2024-02-24

**Authors:** Tao Li, Chao Chen, Lu Liu, Jiapei Qin, Lupeng Qiu, An Wang, Weiwei Dong, Gehan Zhang, Yao Li, Lei Zhao, Fan Zhang, Yi Hu

**Affiliations:** ^1^ Medical School of Chinese PLA Beijing China; ^2^ Department of Oncology, the First Medical Center Chinese PLA General Hospital; Chinese PLA Key Laboratory of Oncology, Key Laboratory for Tumor Targeting Therapy and Antibody Drugs Ministry of Education China; ^3^ Internal Medicine Emergency Department The Second Hospital of Beijing Beijing China; ^4^ Department of Nutrition, The First Medical Center Chinese PLA General Hospital; ^5^ Institute of Translational Medicine, Chinese PLA General Hospital Beijing China

**Keywords:** immunotherapy, modified PD‐1 inhibitors, NSCLC, PD‐1 inhibitors, Pembrolizumab

## Abstract

**Objectives:**

The use of immune checkpoint inhibitors, particularly PD‐1 inhibitors, has revolutionized the treatment of advanced tumors and shown significant improvements in patient survival rates. However, which PD‐1 inhibitor is more effective and safer for a specific indication remains unclear. To address this problem, our study aimed to evaluate the effectiveness and safety of different PD‐1 inhibitors in combination with chemotherapy as first‐line therapy for individuals with advanced non‐small‐cell lung cancer (NSCLC) without driver genes in the real world.

**Materials and Methods:**

We conducted a retrospective study of individuals diagnosed with aNSCLC who received immune checkpoint inhibitors (ICIs) with modified PD‐1 inhibitors, including Sintilimab, Toripalimab, Tislelizumab, Camrelizumab, or Pembrolizumab as first‐line treatment between March 5th, 2016 and October 20th, 2022. We assessed demographic and clinical information and analyzed clinical response, survival outcomes, and safety profiles. The primary endpoint was overall survival (OS), and the secondary endpoints included progression‐free survival (PFS), objective response rate (ORR), disease control rate (DCR), and safety.

**Results:**

As of the date cut‐off on October 20th, 2022, the median follow‐up time was 20.62 months. A total of 204 patients were enrolled in the study, including 56 (27.5%) patients receiving modified PD‐1 inhibitors (Sintilimab, Toripalimab, Tislelizumab, or Camrelizumab) in combination with chemotherapy and 148 (72.5%) patients receiving Pembrolizumab in combination with chemotherapy. In the overall cohort, the median overall survival (OS) was 26.9 months (95%CI, 22.3–31.6), the median progression‐free survival (PFS) was 8.4 months (95%CI, 6.9–9.8), and the objective response rate (ORR) and disease control rate (DCR) were 47.6% (95%CI, 29.9–43.6) and 84.3% (95%CI, 78.4–88.9). The mOS of modified PD‐1 inhibitors group and Pembrolizumab group were 30.7 (95%CI, 17.3–44.4) months and 26.8 (95%CI, 22.2–31.4) months. The mPFS of two groups were 8.3(95%CI, 6.9–9.6) months and 8.8 (95%CI, 6.9–10.7) months, respectively. There was no statistical difference between the two groups in terms of OS or PFS. The ORR for the two groups was 48.2% (95%CI, 34.8–61.8) and 47.3% (95%CI, 39.1–5.6), respectively. However, due to the limited sample size, the difference was not statistically significant. On the other hand, the DCR tended to be higher in the Pembrolizumab group (86.5%; 95%CI, 79.7–91.4) compared to the modified PD‐1 inhibitors group (78.6%; 95%CI, 65.2–87.9), and this difference was statistically significant (*p* = 0.006). In terms of safety, both groups exhibited favorable clinical safety profiles. The only two types of potentially immune‐related adverse events reported were pneumonitis and reactive cutaneous capillary endothelial proliferation (RCCEP).

**Conclusions:**

The modified PD‐1 inhibitors showed comparable survival outcomes and manageable safety profiles in NSCLC compared to Pembrolizumab. Moreover, these inhibitors exhibited improved accessibility and economic outcomes compared to Pembrolizumab. While there were similarities in drug‐related and immunotherapy‐related adverse reactions between the modified PD‐1 inhibitors and Pembrolizumab, there were some slight differences. Further prospective and retrospective studies would be necessary to validate these findings beyond the scope of the CTONG1901 study.

## 1 INTRODUCTION

Cancer is a major public health issue around the world. Lung cancer has become prevalent in China and is the leading cause of cancer‐related deaths in both China and the United States.^1^, ^2^ The US Food and Drug Administration (FDA) and China National Medical Products Administration (NMPA) have approved Pembrolizumab and Nivolumab, two PD‐1 inhibitors based on the KEYNOTE and CheckMate clinical trials, for the treatment of advanced non‐small cell lung cancer (aNSCLC). These approvals have greatly improved the long‐term survival rates of aNSCLC patients.^3^, ^4^ In recent years, modified PD‐1 inhibitors such as Sintilimab,^5^ Toripalimab,^6^ Tislelizumab,^7^ and Camrelizumab,^8^ have been widely used in clinical practice. Some of them have also been listed in the China National Essential Medicare Formulary (NEMF). The use of these drugs in China has increased the availability of immunotherapy medication for cancer patients, thereby improving drug accessibility and economic outcomes.

Currently, comparing the effectiveness and safety of different PD‐1 inhibitors with the same indication poses a significant challenge for both oncologists and patients. The clinical indications for the PD‐1 inhibitors mentioned above were established through direct comparisons with chemotherapy results. However, there is currently limited research data available from retrospective and prospective clinical studies that compare these medications. The initial prospective study CTONG1901, conducted by the Chinese Thoracic Oncology Group (CTONG), showed no substantial differences in safety and efficacy between Sintilimab and Pembrolizumab when used as immune monotherapy or in combination with chemotherapy.^9^

As a result, we analyzed to evaluate the effectiveness and safety of Pembrolizumab and modified PD‐1 inhibitors in combination with chemotherapy as a first‐line treatment for aNSCLC in a real‐world study. This analysis involved reviewing the data of patients treated at the People's Liberation Army General Hospital of China (PLAGH) from its first, third, fourth, fifth, and sixth medical centers over 6 years. Our objective was to provide clinical treatment options supported by data and contribute to further evidence for prospective clinical studies in this area.

## 2 MATERIALS AND METHODS

### 2.1 Study Design and Participants

The objective of this multicenter retrospective cohort study was to investigate the effectiveness and safety of immune checkpoint inhibitors (ICIs), including modified PD‐1 inhibitors and Pembrolizumab, when combined with chemotherapy as first‐line treatment for aNSCLC patients with driver gene negative. The study included confirmed cases of aNSCLC, which encompassed both lung adenocarcinoma and squamous cell carcinoma, in patients with stages IIIA–IV based on the eighth edition of the Union for International Cancer Control (UICC) guidelines.^10^ The study utilized specific criteria for inclusion and exclusion, which are as follows:

1. Patients who had already received a pathological confirmation of lung adenocarcinoma or squamous cell carcinoma with advanced disease or recurrence after surgery.
2. The patients included in this study had complete outpatient and inpatient records, as well as well‐documented long‐term follow‐up records. All of them with drive gene negative, including epidermal growth factor receptor (*EGFR*), anaplastic lymphoma kinase (*ALK*), mesenchymal‐epithelial transformation factor (*MET*), human epidermal growth factor receptor‐2 (*HER‐2*), c‐ros oncogene 1 (*ROS1*), neurotrophin receptor kinase (*NTRK*), RET rearrangements, and vrafmurine sarcoma viral oncogene homolog B (*BRAF*).
3. In this study, patients who received first‐line chemotherapy combined with PD‐1 inhibitors were limited to Pembrolizumab or modified PD‐1 inhibitors (Sintilimab/islelizumab/Toripalimab/Camrelizumab). PD‐1 inhibitors Nivolumab and PD‐L1 inhibitors (Atezolizumab/Durvalizumab/Avelumab/Envafolimab/Sugemalimab) were excluded. It was important to note that all patients included in the study only received PD‐1 inhibitors as their first‐line treatment and did not receive any other types of immune checkpoint inhibitors (PD‐1/PD‐L1/CTLA‐4 inhibitors) in subsequent treatment.

To protect the privacy of the study subjects, deidentified patient data were obtained.

### 2.2 Study objectives

As of the cut‐off date of October 20th, 2022, the median follow‐up time was 20.62 months. The primary endpoint of this study was overall survival (OS), with secondary endpoints including progression‐free survival (PFS), objective response rate (ORR), disease control rate (DCR), and safety. The definition of this study was the time interval from when patients first received PD‐1 inhibitors combined with chemotherapy to death or the last follow‐up. PFS was defined as the time from initial treatment to the first documented disease progression or death. ORR was determined by the proportion of patients with confirmed complete response (CR) or partial response (PR), while DCR was calculated using a combination of confirmed CR, PR, and stable disease (SD). Additionally, the study considered the duration of response and the maximum percentage change from baseline for the sum of diameters of target lesions as secondary endpoints. All endpoints were evaluated according to RECIST guidelines (version 1.1),^11^ and follow‐up imaging reports were independently reviewed by two radiologists. For patients who did not experience disease progression or death events, data were censored at their last follow‐up. Treatment‐related adverse events (TRAEs) and immune‐related adverse events were assessed and graded according to the National Cancer Institute Common Terminology Criteria for Adverse Events (CTCAE, version 5.0).^12^ All patients were followed up until October 20th, 2022, or until their death.

### 2.3 Ethics Approval and Consent to Participate

All participants in this study signed informed consent forms. The ethics committee of PLAGH approved this study according to the ethical standards of the Declaration of Helsinki and its subsequent amendments (Ethical approval number: S2021‐256‐02).

### 2.4 Statistical analysis

The study objectively compared categorical characteristics and response rates between two treatment groups using either the chi‐square or Fisher's exact test. The Mann–Whitney *U* test was used for continuous variables. To assess OS and PFS, Kaplan–Meier survival analysis was employed, with the log‐rank test utilized to compare groups. The Hazard Ratio (HR) was estimated via the Cox proportional hazard model. In the multivariable Cox regression model, factors with a *p*‐value < 0.05 in the univariable Cox regression or deemed clinically relevant were adjusted. All P‐values were two‐tailed, with a *p*‐value < 0.05 indicating statistical significance. Statistical analyses were performed utilizing R (version 4.1.0) and GraphPad Prism (version 9.0). Furthermore, the R package “forest plot” was utilized to create PFS and OS forest plots.

## 3 RESULTS

### 3.1 Patient characteristics and treatment

**FIGURE 1:**
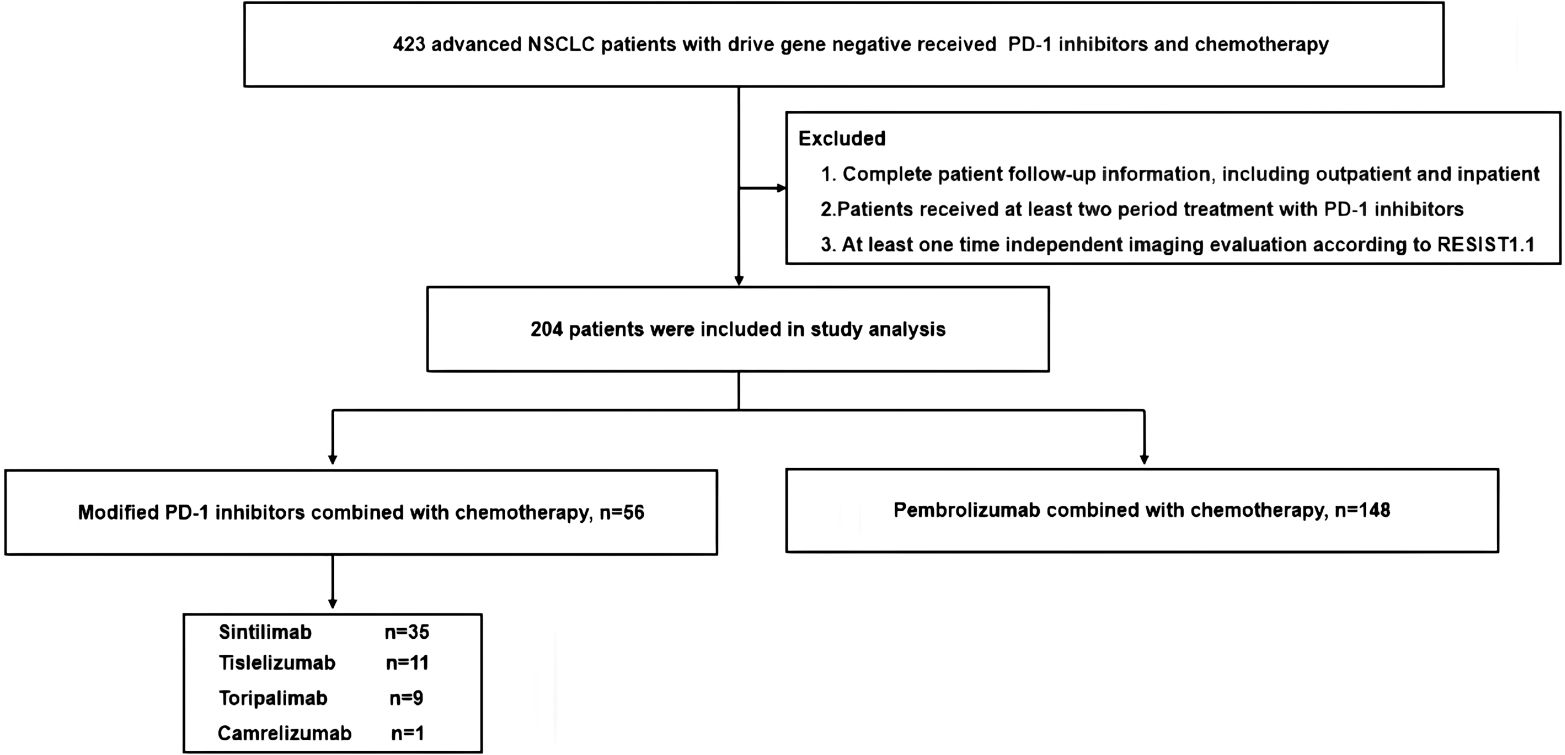
Diagram of this study.

**FIGURE 2:**
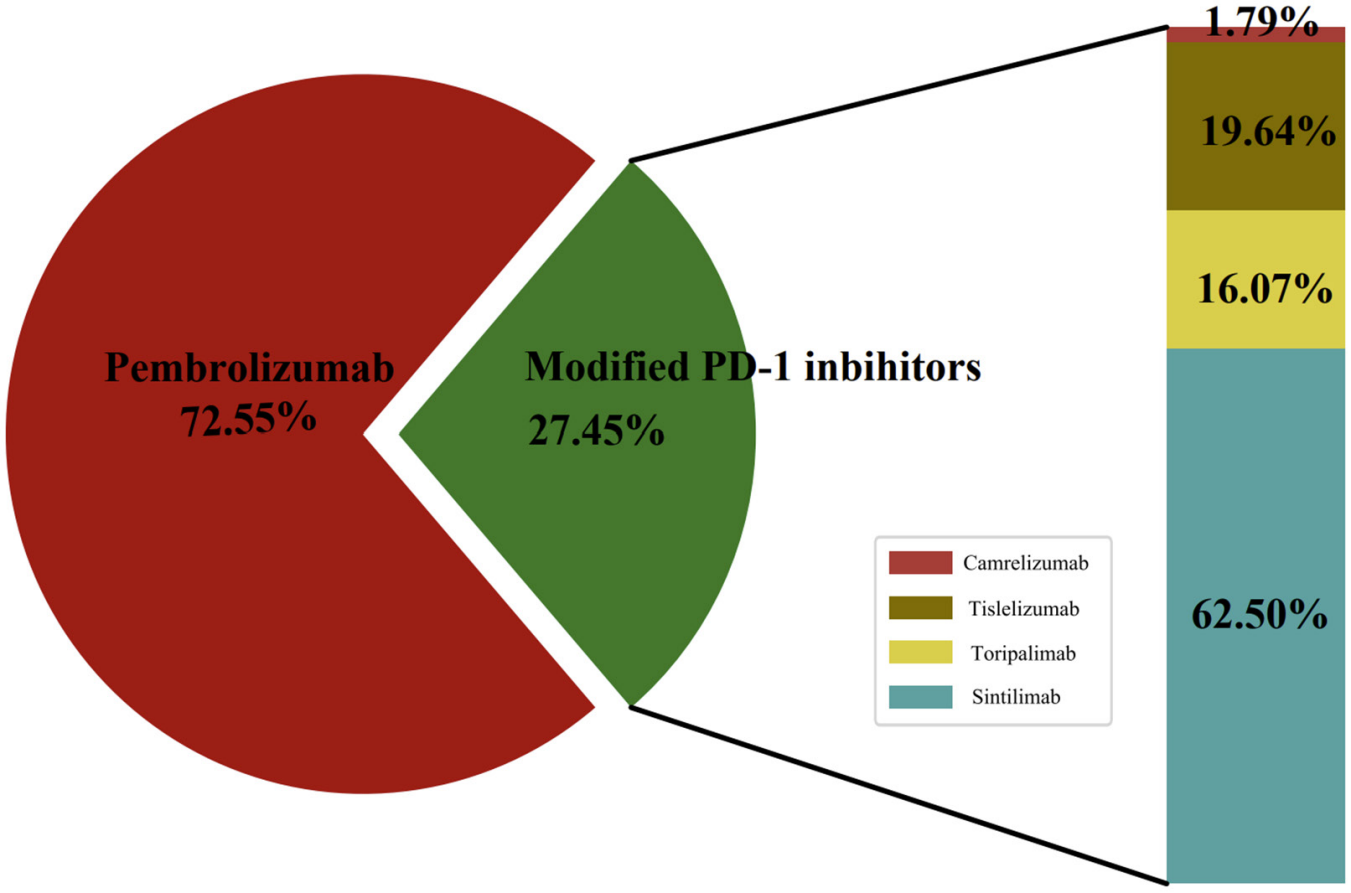
The proportion of modified PD‐1 inhibitors.

From March 5th, 2016, to October 20th, 2022, we assessed 423 patients and finally enrolled 204 patients from five medical centers of PLAGH. Among them, 56 patients (27.5%) received modified PD‐1 inhibitors combined with chemotherapy, and 148 patients (72.5%) received Pembrolizumab combined with chemotherapy. We included all patients who received at least two study treatments with ICIs (modified PD‐1 inhibitors or Pembrolizumab) with chemotherapy (Figures 1 and 2). The chemotherapy regimens primarily consisted of single‐drug or platinum‐based chemotherapy chosen by physicians, including but not limited to platinum drugs, paclitaxel (paclitaxel liposome, albumin‐paclitaxel, docetaxel), gemcitabine, and pemetrexed (for lung adenocarcinoma only). Additionally, based on the PD‐1 inhibitor combined with chemotherapy treatment strategy, some patients received anti‐angiogenesis therapy (Bevacizumab/Anrotinib/Endostar). We provided the baseline demographic and clinical characteristics of the enrolled patients in Table 1. The median age of the patients was 60.3 years, with a median age of 60.9 years for those who received modified PD‐1 inhibitors and 60.1 years for those who received Pembrolizumab. In the modified PD‐1 inhibitors group and the Pembrolizumab group, 51 (91.1%) and 129 (87.2%) patients had a Karnofsky Performance Status (KPS) score of 80–90. The study included four types of modified PD‐1 inhibitors: Sintilimab (35 patients, 62.5%), Tislelizumab (11 patients, 19.6%), Toripalimab (9 patients, 16.1%), and Camrelizumab (1 patient, 1.8%). Regarding treatment, 127 patients (62.3%) received PD‐1 inhibitors combined with chemotherapy, and 77 patients (37.7%) received PD‐1 inhibitors combined with chemotherapy and anti‐angiogenesis therapy. Due to the long duration of the study, people initially did not realize that PD‐L1 status was vital for immunotherapy, so only a part of them completed the PD‐L1 immunohistochemistry (IHC).

**TABLE 1:** Baseline demographic and clinical characteristics.

Parameters	Overall (*N* = 204)	Modified PD‐1 inhibitors group (*N* = 56)	Pembrolizumab (*N* = 148)	*p* Value
Age				0.408
Median, years	60.34 ± 9.19	60.88 ± 10.16	60.14 ± 8.83	
<65	132 (64.7%)	36 (64.3%)	96 (64.9%)	
≥65	72 (35.3%)	20 (35.7%)	52 (35.1%)	
Gender				0.079
Male	162 (79.4%)	49 (87.5%)	113 (76.4%)	
Female	42 (20.6%)	7 (12.5%)	35 (23.6%)	
Smoking history				0.001
Never	63 (30.9%)	10 (17.9%)	53 (35.8%)	
Former	75 (36.8%)	17 (30.3%)	58 (39.2%)	
Current	66 (32.4%)	29 (51.8%)	37 (25.0%)	
Karnofsky Performance status (KPS)				0.439
90–80	180 (88.2%)	51 (91.1%)	129 (87.2%)	
≤**70**	24 (11.8%)	5 (8.9%)	19 (12.8%)	
TNM stage				0.189
III	38(18.7%)	14(25.0%)	25(16.9%)	
IV	165 (81.3%)	42 (75.0%)	123 (83.1%)	
Tumor histology				0.034
Squamous	130(63.7%)	29(51.8%)	101(68.2%)	
Adenocarcinoma	74(36.3%)	27(48.2%)	47(31.8%)	
Histological grade				0.107
I	46(22.5%)	7(12.5%)	39(26.4%)	
II	54 (26.5%)	17 (30.4%)	37 (25.0%)	
III	104 (51.0%)	32 (57.1%)	72 (48.6%)	
PD‐L1 expression				0.326
≥50%	36(17.6%)	7 (12.5%)	29 (19.6%)	
1–49%	43 (21.1%)	14 (25.0%)	29 (19.6%)	
<1%	20(9.8%)	8 (14.3%)	12 (8.1%)	
Unknown	105(51.5%)	27(48.2%)	78(52.7%)	
Hemoglobin, HB				0.133
≥110	164(80.4%)	48(85.7%)	116(78.4%)	
<110	40(19.6%)	8(14.3%)	32(21.6%)	
BMI				0.344
≥18	187(91.7%)	53(94.6%)	134(90.5%)	
<18	17(8.3%)	3(5.3%)	14(9.5%)	
Organs with metastases				<0.001
1	32 (15.7%)	19 (33.9%)	13 (8.78%)	
2	21 (10.3%)	4 (7.14%)	17 (11.5%)	
3	60 (29.4%)	17 (30.4%)	43 (29.1%)	
≥4	91 (44.6%)	16 (28.6%)	75 (50.7%)	
Site of metastases				
Liver	28 (13.7%)	7 (12.5%)	21 (14.2%)	0.754
Bone	67 (32.8%)	20 (35.7%)	47 (31.8%)	0.591
Brain	45 (22.1%)	8 (14.3%)	37 (25.0%)	0.100
Modified PD‐1 type				
Sintilimab		35 (62.5%)		
Tislelizumab		11 (19.6%)		
Toripalimab		9 (16.1%)		
Camrelizumab		1 (1.8%)		
Therapeutic schemes				0.047
Combined with chemotherapy	127(62.3%)	41(73.2%)	86(58.1%)	
Combined with chemotherapy and anti‐angiogenesis therapy	77(37.7%)	15(26.8%)	62(41.9%)	

*Note*: Data are number of patients (%) unless specified otherwise.

### 3.2 Treatment outcomes

**FIGURE 3:**
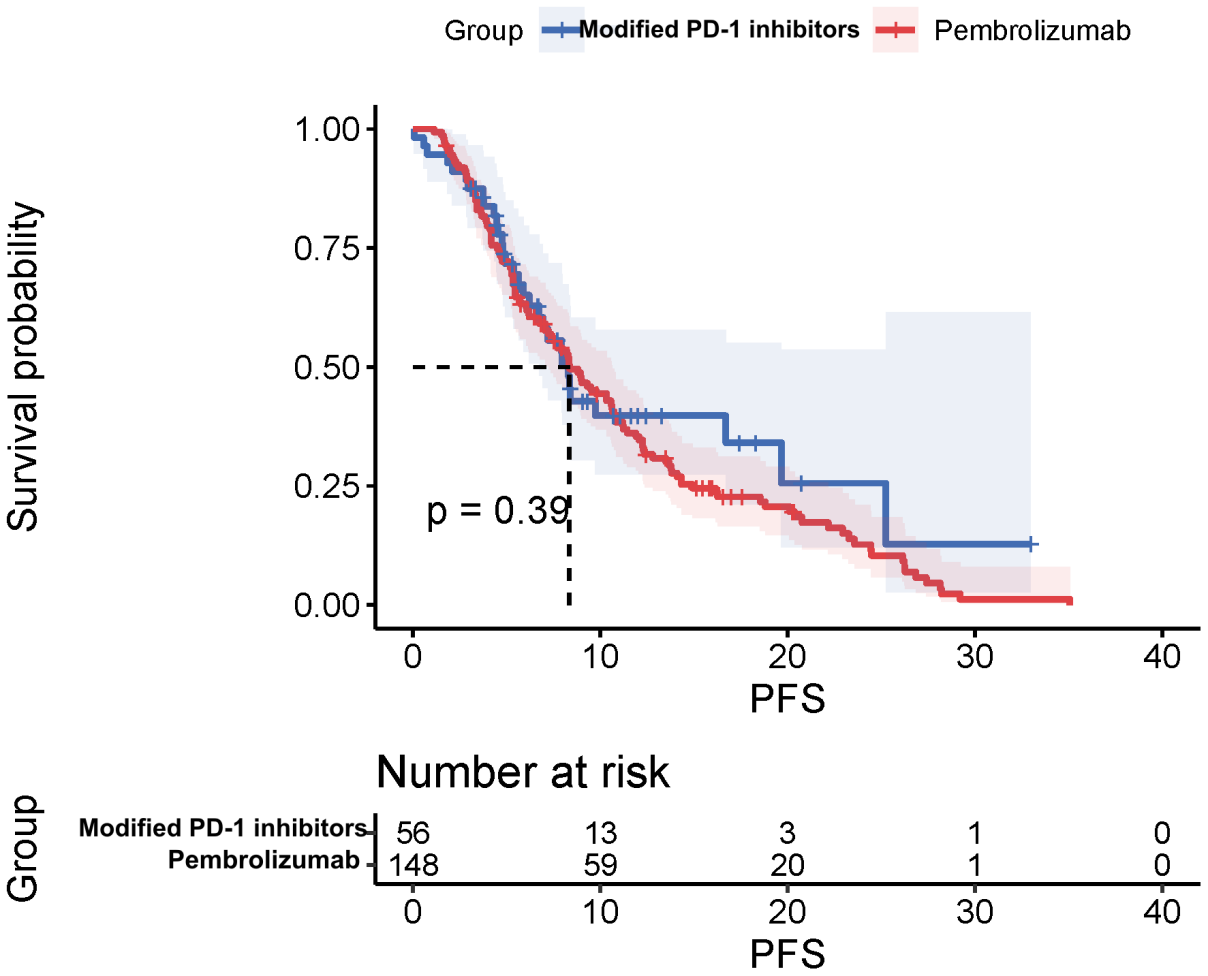
Overall PFS for combination therapy.

**FIGURE 4:**
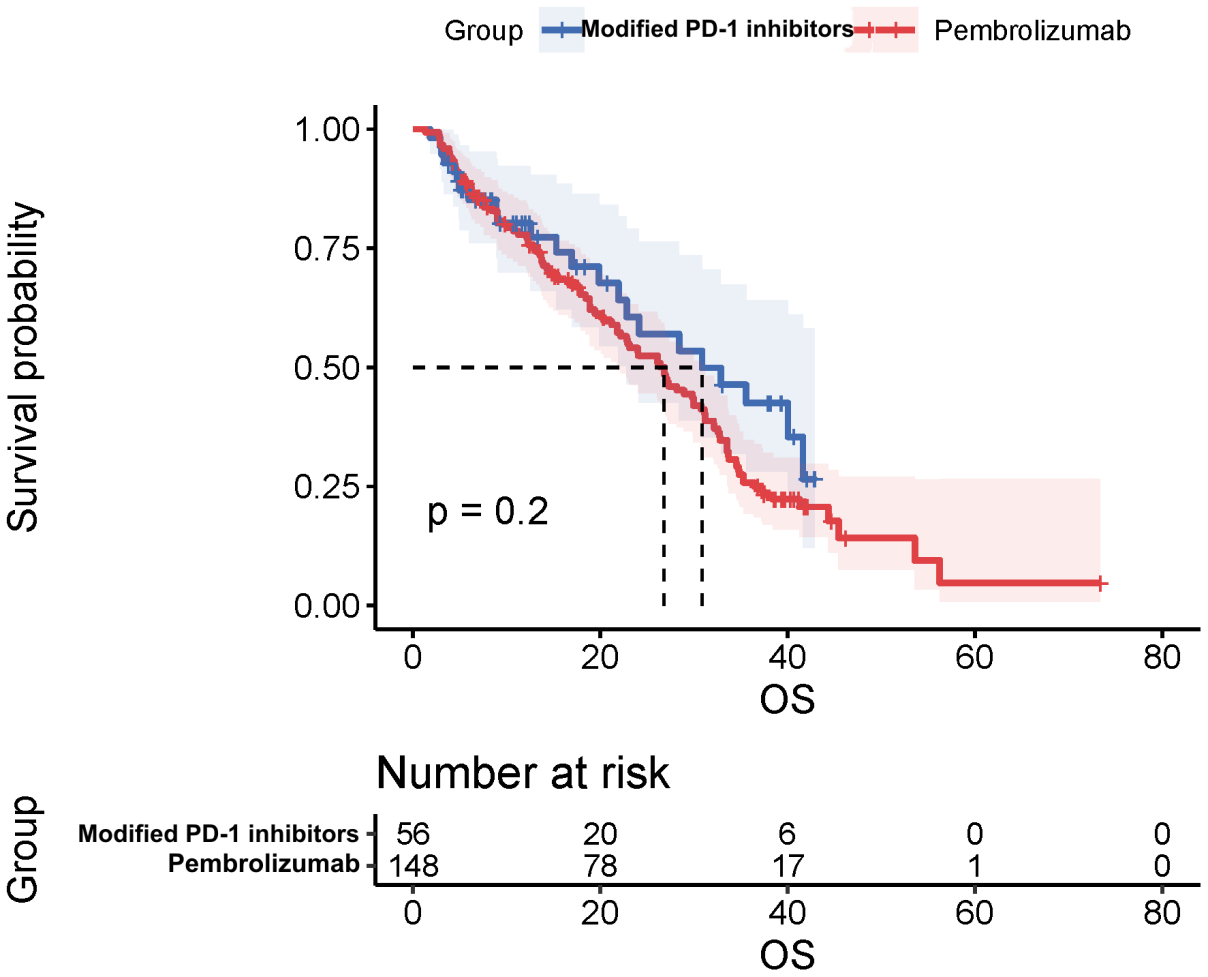
Overall OS for combination therapy.

**FIGURE 5:**
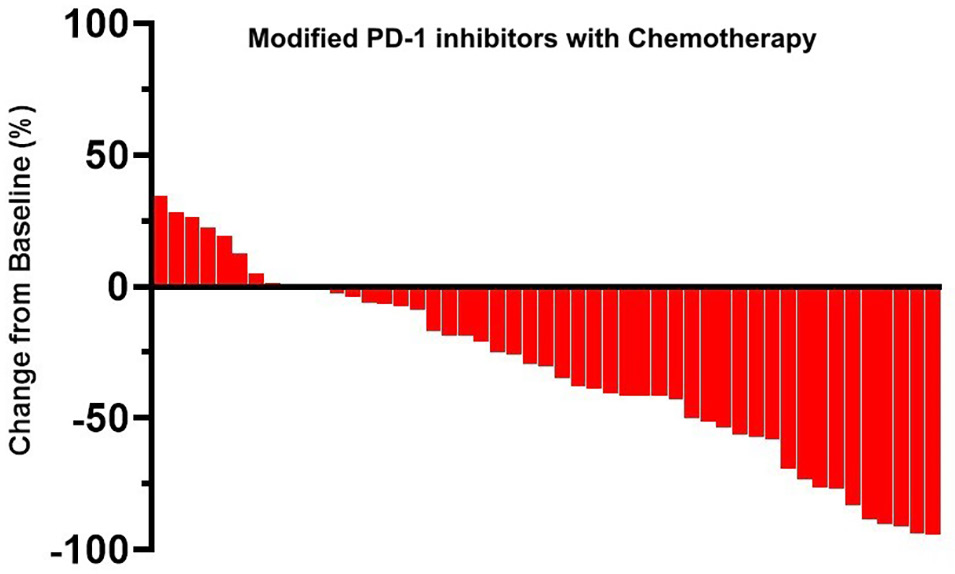
Regression of target lesions from baseline in each patient with modified PD‐1 inhibitors.

**FIGURE 6:**
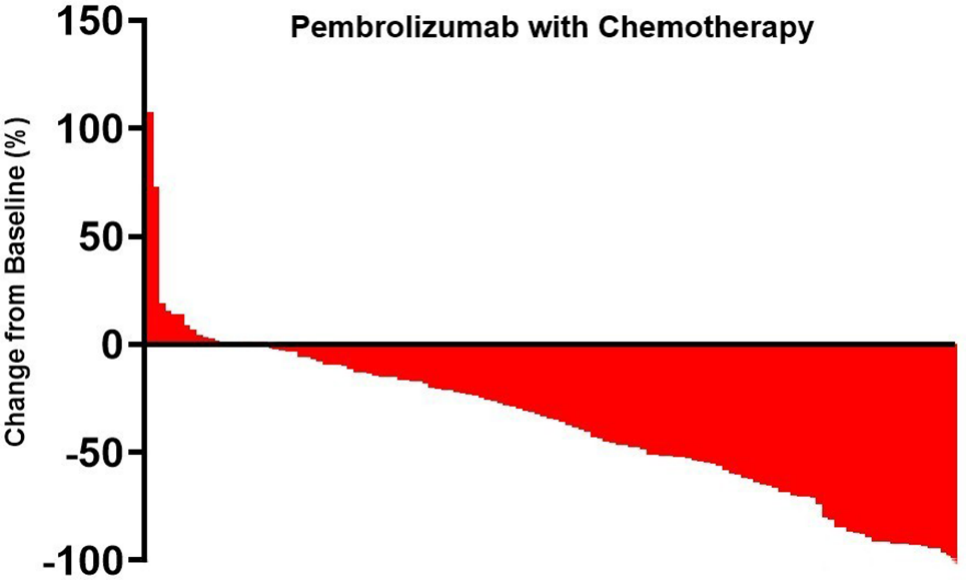
Regression of target lesions from baseline in each patient with Pembrolizumab.

As of the data cut‐off date of October 20th, 2022, the median OS for the entire cohort was 26.9 months (95% CI, 22.3–31.6), and the median PFS was 8.4 months (95% CI, 6.9–9.8) (Figure 3 and Figure 4). The Objective Response Rate (ORR) and Disease Control Rate (DCR) for the overall population were 47.6% (95% CI, 29.9–43.6) and 84.3% (95% CI, 78.4–88.9), respectively (Table 2). The best overall response rates indicated that 27.5% (56/204) of the patients who received modified PD‐1 inhibitors and 72.5% (148/204) of those who received Pembrolizumab treatment experienced a decrease in the sum of their target lesions from baseline (Figures 5 and 6). No statistical difference was found between the two groups in terms of OS or PFS.

**TABLE 2:** Summary of tumor response data.

Tumor response data	Overall population (*N* = 204)	Modified PD‐1 inhibitors (*N* = 56)	Pembrolizumab (*N* = 148)	*p* Values	Modified PD‐1 inhibitors (N = 56)			
					Sintilimab (*N* = 35)	Tislelizumab (*N* = 11)	Toripalimab (*N* = 9)	Camrelizumab (*N* = 1)
Not evaluated	21 (10.3%)	5 (8.9%)	16 (10.8%)		4 (11.4%)	1 (9.1%)	0	0
Complete response (CR)	17 (8.3%)	4 (7.1%)	13 (8.7%)		3 (8.6%)	1 (9.1%)	0	0
Partial response (PR)	80 (39.2%)	23 (41.1%)	57 (38.5%)		14 (40%)	4 (36.4%)	4 (44.5%)	1
Stabe disease (SD)	75 (36.8%)	17 (30.4%)	58 (39.3%)		10 (28.6%)	5 (45.4%)	2 (22.2%)	0
Progressive disease (PD)	11 (5.4%)	7 (12.5%)	4 (2.7%)		4 (11.4%)	0	3 (33.3%)	0
Objective response rate (ORR), 95%CI	97 (47.6%;29.9–43.6)	27 (48.2%;34.8–61.8)	70 (47.3%;39.1–5.6)	0.991	17 (48.6%;31.7–65.7)	5 (45.5%;18.1–75.4)	4 (44.5%;15.3–77.3)	─
Disease control rate (DCR), 95%CI	172 (84.3%;78.4–88.9)	44 (78.6%;65.2–87.9)	128 (86.5%;79.7–91.4)	0.006	27 (77.1%;59.4–88.9)	10 (90.1%;57.1–99.5)	6 (66.7%;30.9–90.9)	─
Median PFS(months,95%CI)	8.4 (6.9–9.8)	8.3 (6.9–9.6)	8.8 (6.9–10.7)	0.445	8.4 (4.9–11.8)	NA	6.2 (3.7–8.6)	─
Median OS(months,95%CI)	26.9 (22.3–31.6)	30.7 (17.3–44.4)	26.8(22.2–31.4)	0.204	30.9 (18.2–43.5)	NA	30.9 (17.2–44.5)	─

*Note*: Data are *n* (%) or *n* (%; 95% CI).

The mOS for the modified PD‐1 inhibitors group was 30.7 months (95% CI, 17.3–44.4), while for the Pembrolizumab group, it was 26.8 months (95% CI, 22.2–31.4). The mPFS for the two groups were 8.3 (95%CI, 6.9–9.6) months and 8.8 (95%CI, 6.9–10.7) months respectively. The ORR of the two groups was 48.2% (95%CI, 34.8–61.8) and 47.3% (95%CI, 39.1–5.6), but the difference was not statistically significant (*p* = 0.991) due to the limited sample size. On the other hand, DCR tended to be higher in the Pembrolizumab group (86.5%; 95%CI, 79.7–91.4) compared to the modified PD‐1 inhibitors group (78.6%; 95%CI, 65.2–87.9), and this difference was statistically significant (*p* = 0.006).

**FIGURE 7:**
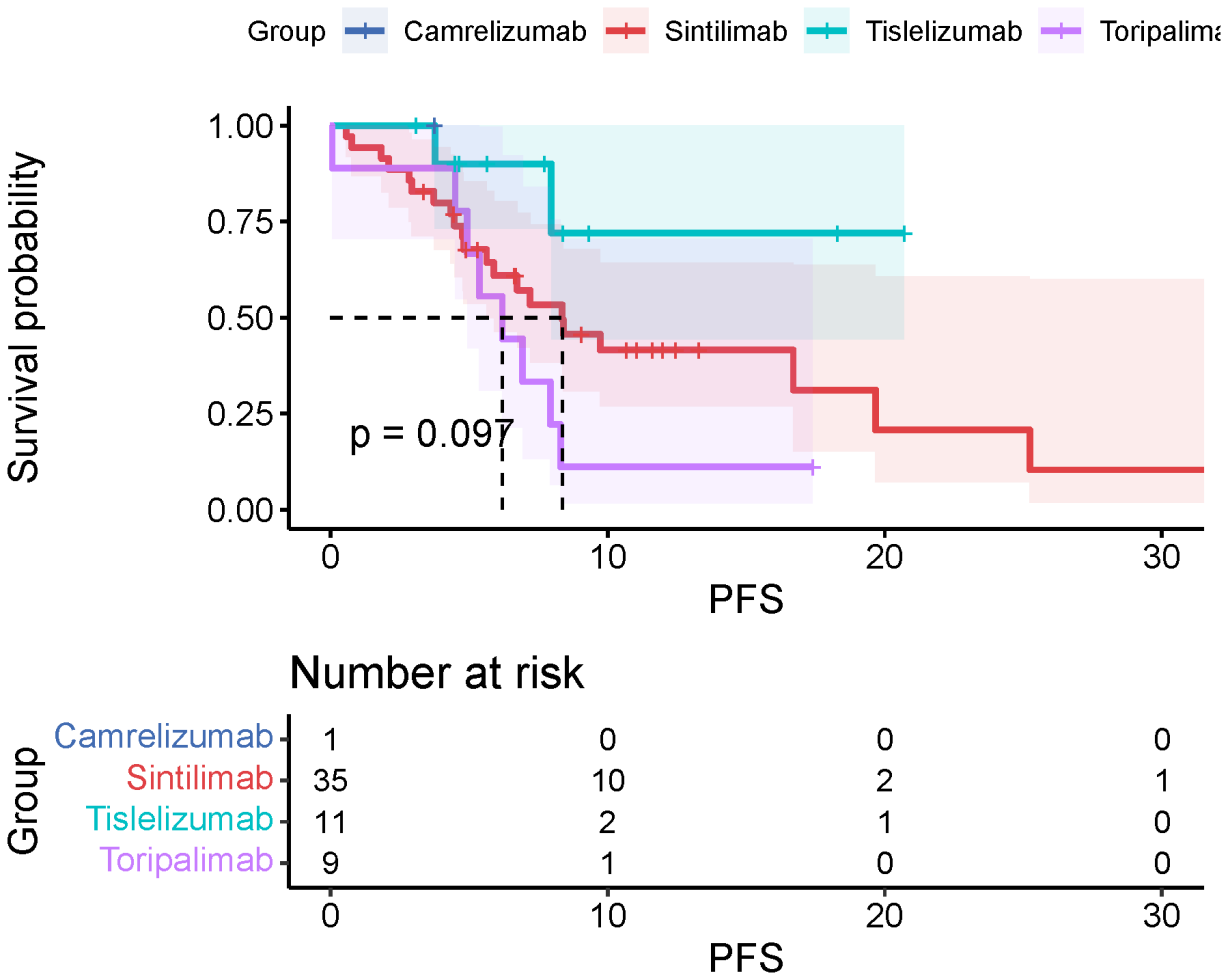
Overall PFS for combination therapy with modified PD‐1 inhibitors.

**FIGURE 8:**
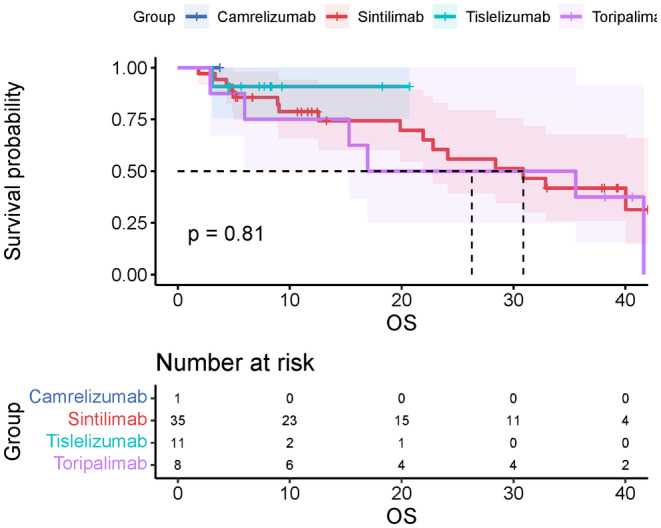
Overall OS for combination therapy with modified PD‐1 inhibitors.

We also conducted a subgroup analysis to compare the survival outcomes of different PD‐1 inhibitors. The mOS of modified inhibitors Sintilimab, Tislelizumab, and Toripalimab were 30.9 m (95%CI, 18.2–43.5), not arrived, 30.9 m (95%CI, 17.2–44.5). The mPFS for them were 8.4 m (95%CI, 4.9–11.8), not arrived, 6.2 m (95%CI, 3.7–8.6). The ORR for Sintilimab, Tislelizumab, and Toripalimab were 48.6% (95%CI, 31.7–65.7), 45.5% (95%CI, 18.1–75.4), and 44.5% (95%CI, 15.3–77.3), respectively. The DCR for different modified inhibitors was 77.1% (95%CI, 59.4–88.9), 90.1% (95%CI, 57.1–99.5), and 66.7% (95%CI, 30.9–90.9). The Kaplan–Meier plot of OS or PFS is shown in Figures 7 and 8.

### 3.3 Association between clinicopathological characteristics and survival outcomes

In univariate and multivariate analyses using the Cox regression model, we selected several factors to analyze their impact on patient prognosis. These factors included the patient's primary condition, physical status, tumor heterogeneity, treatment strategies, and treatment lines. More detailed information about these factors and their influence on patient prognosis can be found in Tables 3 and 4.

**TABLE 3:** Univariable and multivariable analysis of PFS.

Parameters	Univariable analysis			Multivariable analysis		
	HR	95%CI	*p* Values	HR	95%CI	*p* Values
Gender						
Male vs Female	1.267	(0.801–2.003)	0.311			
Age						
<65 vs. >65	1.269	(0.829–1.943)	0.273			
Smoking status						
Former or Current vs. Never	1.063	(0.704–1.604)	0.773			
Tumor histology						
Adenomatous vs. Sequamous	0.744	(0.491–1.128)	0.164			
Histological grade						
III vs. II	1.046	(0.666–1.641)	0.847			
III vs. I	0.573	(0.344–0.955)	0.033			
PD‐L1						
Positive vs. negative	1.424	(0.947–2.143)	0.090			
HB						
≥110 vs. <110	1.494	(0.954–2.339)	0.079			
LDH level at baseline						
≥250 vs. <200	0.488	(0.318–0.747)	<0.001	0.573	(0.363–0.904)	0.017
Metastatic site						
Liver						
No vs Yes	1.857	(1.147–3.007)	0.012	1.404	(0.835–2.360)	0.201
Bone						
No vs. Yes	1.240	(0.827–1.861)	0.298			
Brain						
No vs. Yes	1.356	(0.878–2.092)	0.169			
Anti‐PD‐1 agents						
Modified made PD‐1 vs. Pembrolizumab	0.778	(0.483–1.251)	0.300			
Anti‐angiogenic therapy						
No vs. Yes	1.481	(1.005–2.181)	0.047	1.184	(0.777–1.803)	0.433

**TABLE 4:** Univariable and multivariable analysis of OS.

Parameters	Univariable analysis			Multivariable analysis		
	HR	95%CI	*p* Values	HR	95%CI	*p* Values
Gender						
Male vs Female	0.874	(0.596–1.283)	0.492			
Age						
<65 vs ≥65	1.166	(0.832–1.635)	0.373			
Smoking status						
Former or Current vs. Never	0.953	(0.678–1.339)	0.78			
Tumor histology						
Adenomatous vs. Sequamous	1.146	(0.818–1.605)	0.429			
Histological grade						
III vs. II	1.517	(1.028–2.238)	0.036	1.373	(0.927–2.033)	0.113
III vs. I	1.552	(1.061–2.272)	0.024	1.447	(0.985–2.127)	0.06
PD‐L1						
Positive vs. negative	1.229	(0.892–1.693)	0.208			
HB						
≥110 vs. <110	1.322	(0.899–1.944)	0.156			
LDH level at baseline						
≥250 vs. <200	0.7	(0.484–1.014)	0.059			
Metastatic site						
Liver						
No vs. Yes	1.135	(0.722–1.784)	0.583			
Bone						
No vs. Yes	0.849	(0.604–1.193)	0.345			
Brain						
No vs. Yes	1.141	(0.791–1.648)	0.48			
Anti‐PD‐1 agents						
Modified PD‐1 inhibitors vs. Pembrolizumab	1.209	(0.814–1.795)	0.348			
Anti‐angiogenic therapy						
No vs. Yes	1.217	(0.885–1.671)	0.227			

**FIGURE 9:**
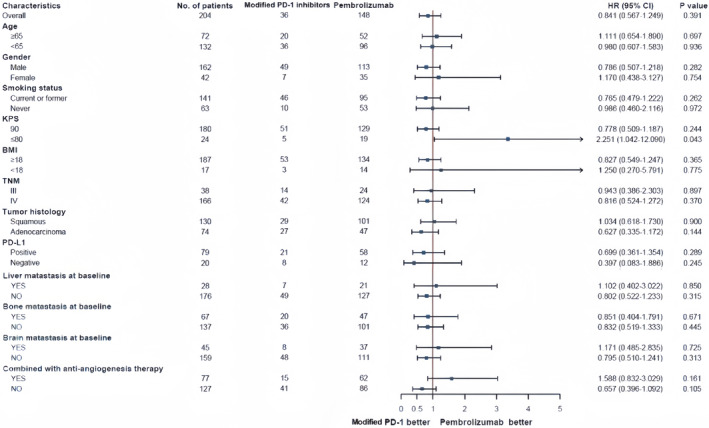
Forest plot of hazard ratios for PFS.

Based on the results from the univariate analyses, we selected higher histological grade and more prior lines for metastatic disease as factors for further multivariable analyses in terms of PFS. The hazard ratios (HR) for higher histological grade were 1.517 (95% CI, 1.028–2.238) and 1.552 (95% CI, 1.061–2.272), indicating that patients with a higher histological grade had a higher risk of disease progression. Similarly, the HR for prior lines for metastatic disease was 2.085 (95% CI, 1.518–2.866), suggesting that patients with more prior lines for metastatic disease were also at a higher risk of disease progression. After conducting multivariable analyses, only the number of prior lines for metastatic disease remained as the independent risk factor associated with PFS outcomes in patients receiving PD‐1 inhibitors combined with chemotherapy. The HR was 1.997 (95% CI, 1.448–2.753, *p* < 0.001), indicating that each additional prior line for metastatic disease increased the risk of disease progression. This result is illustrated in Figure 9.

**FIGURE 10:**
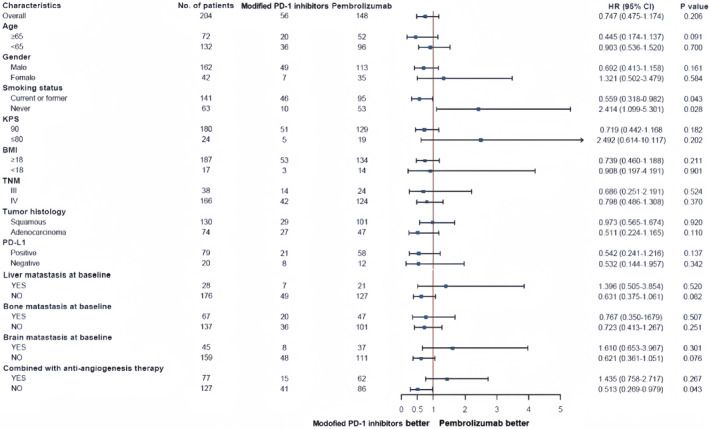
Forest plot of hazard ratios for OS.

In terms of OS, initial treatment for patients with lactic dehydrogenase, LDH≥250 U/L, more prior lines for metastatic disease, liver metastases, and PD‐1 inhibitors combined with chemotherapy and anti‐angiogenic therapy were at higher risk of death HR for LDH level at baseline was 0.488 (95%CI,0.318–0.747); HR for prior lines for metastatic disease was 1.585 (95%CI,1.073–2.342); HR for liver metastases was 1.857 (95%CI,1.147–3.007); HR for PD‐1 combined with chemotherapy and anti‐angiogenic therapy was 1.481 (95%CI,1.005–2.181). Finally, high LDH level (≥250 U/L) was the only independent risk factor associated with OS outcomes in patients with PD‐1 inhibitors combined with chemotherapy. Prior lines for metastatic disease, liver metastases, and PD‐1 inhibitors combined with chemotherapy and anti‐angiogenic therapy did not reach statistical significance in the multivariable analysis (Figure 10).

### 3.4 Safety and adverse events

We present a detailed statistical summary of adverse reactions in patients who received ICIs combined with chemotherapy. Table 5 shows TRAEs of any grade occurring in the overall cohort. Among the overall cohort, the following all‐grade treatment‐related adverse events (TRAEs) were observed in 5% or more of the patients: decreased appetite, decreased white blood cell count and neutrophil count, asthenia, nausea, increased aspartate aminotransferase (AST) and alanine aminotransferase (ALT), vomiting, alopecia, anemia, increased blood bilirubin, rash, diarrhea, increased blood creatinine, and pruritus. Eight patients (3.9%) experienced grade 3–4 events, including decreased white blood cell count, neutrophil count, and pneumonitis. Within the cohort, 17 patients experienced potentially immune‐related adverse events. Among them, pneumonitis and reactive cutaneous Capillary endothelial proliferation (RCCEP) were reported. Four cases of RCCEP were observed in the modified PD‐1 inhibitors group, while 13 cases were observed in the Pembrolizumab group.

**TABLE 5:** Safety and adverse events in the overall population.

Parameters	Overall (*N* = 204)	Modified PD‐1 inhibitors (*n* = 56)			Pembrolizumab (*n* = 148)		
		Any grade	Grade 3–4	Death	Any grade	Grade 3–4	Death
Any TRAEs							
Decreased appetite	87 (62.6)	23	0	0	64	0	0
White blood cell count decreased	106 (52.0)	28	2 (3.6)	0	78	3 (2.0)	0
Neutrophil count decreased	99 (48.5)	26	1 (1.8)	0	73	2 (1.4)	0
Asthenia	84 (41.2)	22	0	0	62	0	0
Nausea	48 (23.5)	12	0	0	36	0	0
AST increased	33 (16.2)	7	0	0	26	0	0
ALT increased	30 (14.7)	8	0	0	22	0	0
Vomiting	27 (13.2)	8	0	0	19	0	0
Alopecia	20 (9.8)	6	0	0	14	0	0
Anemia	16 (7.8)	4	0	0	12	0	0
Blood bilirubin increased	14 (6.9)	2	0	0	12	0	0
Rash	13 (6.4)	6	0	0	7	0	0
Diarrhea	13 (6.4)	6	0	0	7	0	0
Blood creatinine increased	13 (6.4)	3	0	0	10	0	0
Pruritus	11 (5.4)	3	0	0	8	0	0
Hypoalbuminemia	5 (2.5)	3	0	0	2	2 (1.4)	0
Mucositis oral	3 (1.5)	1	0	0	2	0	0
Hypothyroidism	0	0	0	0	0	0	0
Additional TRAEs of special interest							
CIP	17 (8.3)	4	1(Sintilimab)	1(Tislelizumab)	13	2 (1.4)	0
RCCEP	1 (0.5)	1(Camrelizumab)	0	0	0	0	0

*Note*: Data are number of patients (% of patients).

Abbreviations: ALT, alanine aminotransferase; AST, alkaline phosphatase; CIP, Checkpoint Inhibitor Pneumonitis; RCCEP, Reactive Cutaneous Capillary Endothelial Proliferation; TRAEs, treatment‐related adverse events.

In the Pembrolizumab group, there were no deaths related to the study treatment. However, one death was attributed to treatment in the modified PD‐1 inhibitors group due to pneumonitis (specifically associated with Tislelizumab), and grade 4 pneumonitis occurred as a result of Sintilimab treatment. Furthermore, this study also observed RCCEP,^13^ a specific immunotherapy‐related adverse reaction to Camrelizumab. However, the adverse effect was reported to be mild according to related studies.

In general, the clinical safety of PD‐1 inhibitors, whether modified PD‐1 inhibitors or Pembrolizumab combined with chemotherapy, was favorable. The occurrence of drug‐related and immunotherapy‐related adverse events remained at a low level. Importantly, these adverse events were found to be preventable and manageable through appropriate monitoring and intervention strategies.^14^, ^15^

## 4 DISCUSSION

In the past, there was a perception that medications produced by Chinese biopharmaceutical enterprises, especially anti‐tumor medications, were inferior to those developed by foreign companies. As a result, the public generally had a traditional concept that foreign drugs must be superior to those produced by Chinese enterprises. Chinese medications were often considered equivalent to generic medications. However, with the advancement of medical science and technology research in China, there has been a shift towards greater emphasis on the research and development of innovative drugs by Chinese biopharmaceutical enterprises. In the field of tumor immunotherapy, specifically ICIs, China has made significant progress. Apart from well‐known Pembrolizumab, Nivolumab, Avelumab, Atezolizumab, and Durvalizumab developed by large multinational companies, there are now at least 10 modified PD‐1/PD‐L1 inhibitors that have been developed and produced by Chinese companies and approved by NMPA in mainland China. These included Sintilimab, Tislelizumab, Toripalimab, Camrelizumab, Envolimab, Putlimab, and others. Some of these medications have been included in NEMF, which has greatly improved the accessibility and affordability of therapy for patients. This expansion of treatment options has increased patient satisfaction and met the demand for cancer medications.^16^, ^17^, ^18^ Details of the time of approval, indications, and other relevant information were provided in Table S2.

The complexity of drug modification, including issues related to structure, function, and effects, often involves trade secrets and patent ownership. As a result, it could be challenging to determine the theoretically better clinical effect of different modified PD‐1 inhibitors based on publicly available information. In clinical practice, oncologists and patients are often faced with the dilemma of choosing among various PD‐1/PD‐L1 inhibitors that have similar indications but whose differences in effectiveness and safety remain unclear. To address this issue, the team of Professor Yilong Wu from Guangdong Provincial People's Hospital initiated the first head‐to‐head prospective comparison study between Pembrolizumab and Sintilimab. CTONG1901 study was an open‐label, randomized controlled phase II clinical study aimed to evaluate the effectiveness and safety of single‐agent or combination chemotherapy with Pembrolizumab or Sintilimab in untreated patients with aNSCLC with driver gene‐negative. Relevant study information was gradually published in the American Society of Clinical Oncology (ASCO) 2021 and the World Lung Cancer Congress (WCLC) 2022. The median follow‐up time was 11.5 (0.7–25) months. The relevant treatment results of the Sintilimab group (single agent or combined chemotherapy) and the Pembrolizumab group (single agent or combined chemotherapy) were shown in the Table S1. This prospective study demonstrated no significant difference in effectiveness and safety between Sintilimab and Pembrolizumab, as assessed by primary endpoint ORR and related secondary endpoints DCR, PFS, and OS. The anti‐tumor activity was found to be similar, and the adverse reactions were consistent with previous study reports. Furthermore, no new adverse reactions were identified, and the study is still ongoing. Although the sample size of this study is small, the results provide valuable guidance and insights for clinical practice in terms of head‐to‐head comparisons of different PD‐1 inhibitors.^9^

Currently, several modified PD‐1/PD‐L1 inhibitors have advantages over imported agents (Pembrolizumab or Nivolumab) in clinical applications. These advantages can be attributed to their specific structural modifications and functional characteristics:

### 4.1 Structural advantages of modified anti‐tumor PD‐1 antibodies

Sintilimab exhibits strong and specific binding to human and cynomolgus PD‐1, with an affinity to human PD‐1 measured at 0.3 nM via surface SPR and slow dissociation kinetics. Additionally, Sintilimab blocked the interaction between PD‐1 and both PD‐L1 and PD‐L2, leading to high secretion levels of both interferon (IFN)‐γ and interleukin (IL)‐2 in primary T cell assays.^19^, ^20^

Tislelizumab modified the Fc segment to remove the binding ability of the FC‐γ receptor on the surface of macrophages. This modification helps avoid the antibody‐dependent cellular phagocytosis (ADCP) induced by macrophages, thereby protecting the functional activity of T cells and theoretically enhancing their effectiveness^21^, ^22^ (Figure S1).

Envafolimab was a single‐domain antibody Fc fusion protein targeting PD‐L1.^23^ Compared with the structure of two light chains and two heavy chains of conventional human antibodies, the antigen‐binding region composed only by the heavy chain was a single domain connected by the hinge region and the Fc region, which has strong stability and still had the function of binding antigen after isolation on the autoantibody.^24^

These structural modifications enhance the binding specificity, stability, and functional activities of these modified PD‐1/PD‐L1 inhibitors, potentially improving their effectiveness in tumor immunotherapy.

### 4.2 Usage characteristics

One notable feature of Envafolimab is its subcutaneous injection formulation.^25^ This formulation greatly improves patients' compliance with treatment, particularly during the COVID‐19 epidemic, as it eliminates the need for repeated hospital intravenous infusions and reduces the risk of infection.

### 4.3 The economic advantages

Among the modified PD‐1 inhibitors, four have been included in NEMF, and four have clear charity assistance policies. Whether included in NEMF or covered by charity assistance, these medications have shown a significant reduction in total costs and average costs for patients compared to imported PD‐1 inhibitors. These cost reductions have increased the accessibility of these medications, especially at county‐level hospitals.^17^, ^18^, ^26^

It is important to note that these advantages do not indicate that modified medications have completely surpassed imported ones. There are still areas that require improvement in terms of drug structure, clinical research, and clinical application of modified PD‐1 inhibitors.

#### 4.3.1 Lack of differentiation among modified PD‐1 inhibitors

The field of PD‐1/PD‐L1 inhibitors has been revolutionary in the development of innovative anti‐tumor medications. These inhibitors have demonstrated superior effectiveness, safety, and a wide range of indications. However, currently available PD‐1/PD‐L1 inhibitors on the market suffer from significant homogeneity, with limited differentiation. Many of these medications are simply the accumulation of quantity rather than introducing important breakthroughs, which has led to the term “PD‐X". It is worth noting that there have been approximately 3250 registered clinical trials worldwide for PD‐1/PD‐L1 inhibitors, with 817 listed as Phase 3 clinical trials.^27^ In China alone, there were over 657 registered clinical trials on PD‐1/PD‐L1 inhibitors involving more than 150 enterprises, with 183 in the Phase 3 clinical stage. This lack of product differentiation and excessive duplication of studies highlighted the need for biopharmaceutical companies to accelerate the development of new immunotherapy medications, explore new indications for existing medications, and expand the scope of drug combination strategies.

#### 4.3.2 Lack of reliable clinical research results

Despite the production of multiple PD‐1/PD‐L1 inhibitors, there was a lack of more robust clinical research that included a diverse population and covered various cancer types. Recently, modified PD‐1 inhibitors such as Sintilimab and Tislelizumab applied for FDA approval for marketing in the United States but were refused approval. This refusal was primarily due to the insufficient number of patients included in the trials, which did not adequately represent the U.S. population. Conducting clinical trials of innovative medications overseas was still in the exploratory stage, and these two failed cases highlighted the mature market's concerns regarding the quality of clinical trials conducted in China. Neither of these modified PD‐1 products had sufficient data from U.S. patients in their clinical trials, and there were no head‐to‐head comparisons conducted with existing medications, particularly Pembrolizumab.

#### 4.3.3 Unique adverse reactions of certain medications

It was important to pay attention to the unique adverse reactions associated with specific medications. For example, Camrelizumab has been associated with a unique adverse reaction known as Reactive Cutaneous Capillary Endothelial Proliferation (RCCEP).^13^, ^28^ The incidence of RCCEP with Camrelizumab is approximately 77.4%, but most cases were grade 1–2 adverse reactions. Grade 3 or higher RCCEP occurs in only 1.1% of cases, and there have been no reports of grade 4 adverse events or deaths related to RCCEP. RCCEP typically occurs around 1 month after injection and follows a self‐limited course, resolving gradually within 6 months. So far, RCCEP has only been observed in the epidermis and skin during clinical practice. These unique adverse reactions should be closely monitored and managed during treatment with Camrelizumab.

In clinical practice, modified PD‐1/PD‐L1 inhibitors are commonly used in combination with chemotherapy as first‐line treatment for NSCLC. However, it was important to note that no monotherapy regimen of these inhibitors had been approved for NSCLC. Additionally, the use of modified PD‐1/PD‐L1 inhibitors as neoadjuvant therapy or postoperative adjuvant therapy had not yet received approval. Currently, Pembrolizumab and Nivolumab are the only two PD‐1 inhibitors that have long‐term survival data of up to 5 years data.^4^, ^29^ These two inhibitors had gained approval for a wide range of oncology indications, including neoadjuvant therapy, postoperative adjuvant therapy, and advanced therapy. Their approval in various indications reflected their established efficacy and safety profiles in multiple clinical scenarios.

This study represented the first retrospective analysis reporting on the effectiveness and safety of different modified PD‐1 inhibitors. The findings further support the initial findings from the CTONG1901 trial, indicating that modified PD‐1/PD‐L1 inhibitors had similar efficacy compared to imported ones and that adverse reactions could be effectively prevented and controlled. However, it was important to acknowledge the limitations of this retrospective study. The overall sample size and follow‐up time were limited, which may have impacted the robustness of the results. Additionally, the study focused only on four specific modified PD‐1 inhibitors due to their availability and launch time in China. To enrich the findings, future studies should aim to expand the sample size, extend the follow‐up duration, and include a wider range of PD‐1 medications.

Indeed, the goal of comparing different modified PD‐1 inhibitors was not merely to establish superiority or inferiority but rather to promote and enhance drug research, development, and innovation. By conducting such comparisons, we aimed to provide valuable information that could assist oncologists in selecting more appropriate treatment options for their cancer patients. Moving forward, it was anticipated that there would be a continued focus on the development of more modified PD‐1 inhibitors with enhanced effectiveness. This ongoing research and innovation hold the potential to further benefit cancer patients by expanding treatment options and improving outcomes.

Additionally, as Chinese‐produced PD‐1 inhibitors continued to demonstrate their efficacy and safety, it was hoped that these medications would gain recognition and acceptance internationally. The objective was to contribute to global efforts in combating cancer and to offer more treatment options to cancer patients worldwide.

## AUTHOR CONTRIBUTIONS

**Tao Li:** Formal analysis (equal); writing – original draft (equal); writing – review and editing (equal). **Chao Chen:** Formal analysis (equal); methodology (equal). **Lu Liu:** Conceptualization (equal); resources (equal). **Jiapei Qin:** Investigation (equal); methodology (equal). **An Wang:** Methodology (equal); resources (equal). **Yao Li:** Data curation (equal); formal analysis (equal). **Lupeng Qiu:** Methodology (equal); project administration (equal). **Weiwei Dong:** Data curation (equal); formal analysis (equal). **Gehan Zhang:** Project administration (equal); software (equal). **Lei Zhao:** Validation (equal); visualization (equal). **Fan Zhang:** Software (equal); supervision (equal); writing – review and editing (equal). **Yi Hu:** Supervision (equal); writing – review and editing (equal).

## FUNDING INFORMATION

This research did not receive any specific grant from funding agencies in the public, commercial, or not‐for‐profit sectors.

## CONFLICT OF INTEREST STATEMENT

The authors declare that the research was conducted without any commercial or financial relationships that could be construed as a potential conflict of interest.

## ETHICS STATEMENT

All participants in this study signed informed consent forms. The ethics committee of the PLA General Hospital of China approved this study according to the ethical standards of the Declaration of Helsinki and its subsequent amendments (Ethical approval number: S2021‐256‐02).

## Supporting information


Figure S1.

Table S1.

Table S2.

## Data Availability

Data are available upon reasonable request. The data supporting this study's findings are available from the corresponding author upon reasonable request.
