# Supplementary figures and images for: A multicenter, real‐world study on effectiveness and safety of first‐line modified PD‐1 inhibitors with chemotherapy in advanced non‐small cell lung cancer (aNSCLC) with drive gene‐negative
Source: Cancer Med. 2024 Feb 24;13(3):e7024. doi: 10.1002/cam4.7024 (PMC10891446; doi:10.1002/cam4.7024)

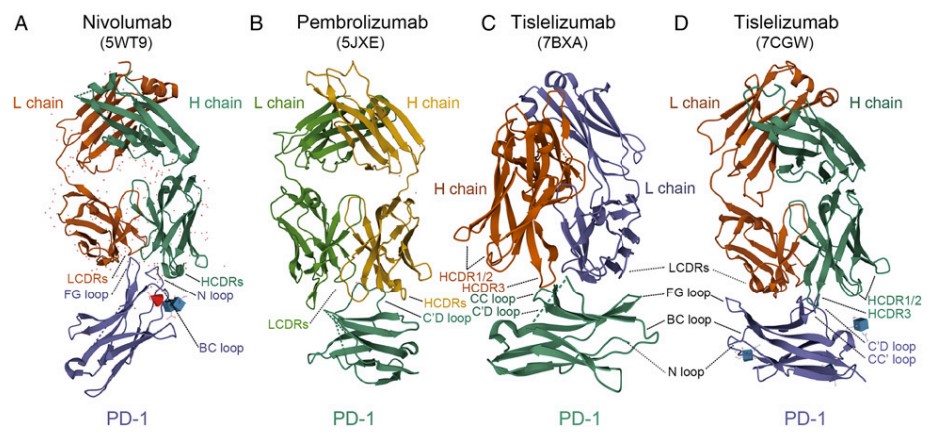

Supplement: Supplementary file 1 — Figure S1. [file CAM4-13-e7024-s003.jpg]
